# Photobiomodulation Therapy Does Not Attenuate Fatigue and Muscle Damage in Judo Athletes: A Randomized, Triple-Blind, Placebo-Controlled Trial

**DOI:** 10.3389/fphys.2019.00811

**Published:** 2019-06-26

**Authors:** Lucas Bet da Rosa Orssatto, Daniele Detanico, Rafael Lima Kons, Raphael Luiz Sakugawa, Jorge Nelson da Silva, Fernando Diefenthaeler

**Affiliations:** ^1^School of Exercise and Nutrition Sciences, Institute of Health and Biomedical Innovation, Faculty of Health, Queensland University of Technology, Brisbane, QLD, Australia; ^2^Laboratório de Biomecânica, Departamento de Educação Física, Universidade Federal de Santa Catarina, Florianópolis, Brazil

**Keywords:** combat sports, recovery, performance, low-level laser therapy, stretch shortening cycle, photobiomodulation

## Abstract

Fatigue and muscle damage negatively affect performance in lower limb exercises involving the stretch-shortening cycle in judo athletes during competition and training sessions. Photobiomodulation therapy has emerged as an effective non-invasive strategy to attenuate fatigue and muscle damage when applied before different types of exercises. Our objective was to investigate the effects of photobiomodulation therapy on fatigue and muscle damage in judo athletes. Sixteen judo athletes participated in the study (23.1 ± 3.8 years, 77.9 ± 14.9 kg, 173.1 ± 8.9 cm, 17.5 ± 7.3 body fat%, 12.9 ± 5.0 years of practice). Each participant received, in a randomized manner, photobiomodulation in one limb and placebo in the contralateral limb on the same day. Thereafter, subjects performed a stretch-shortening cycle protocol to induce muscle fatigue and damage. Countermovement jump (impulse, peak power, peak velocity, and peak force), echo intensity (*rectus femoris* and *vastus lateralis*), and muscle soreness were assessed at different time points before, during, immediately post, and 24 and 48 h after the protocol. Muscle fatigue was detected due to reductions in countermovement jump impulse (14.7 ± 9.8 and 15.9 ± 15.5%), peak power (12.9 ± 8.5 and 11.9 ± 6.9%), peak velocity (8.6 ± 8.1 and 6.5 ± 6.0%), and peak force (7.0 ± 5.3 and 8.0 ± 6.1%) after the protocol (*p* < 0.001), for placebo and photobiomodulation therapy, respectively. Muscle damage was detected due to reductions in countermovement jump impulse (−6.1 ± 19.2% and −4.5 ± 9.2%, *p* < 0.05), increases in echo intensity (*rectus femoris*, 21.0 ± 11.9 and 20.8 ± 9.0%; and *vastus lateralis*, 22.4 ± 23.2%; and 16.7 ± 23.8%; *p* < 0.001), and *quadriceps* muscle soreness (3.6 ± 1.6 and 3.5 ± 1.7 a.u; *p* < 0.011), 48 h after the protocol, for placebo and photobiomodulation therapy, respectively. No differences were observed between photobiomodulation therapy and placebo at any time points for any variables (*p* > 0.05), indicating no positive effect favoring photobiomodulation therapy. In conclusion, our findings suggest no effect of photobiomodulation therapy applied before exercise to reduce lower limb muscle fatigue and damage during and following a stretch-shortening cycle protocol in judo athletes.

## Introduction

In an official judo competition, athletes perform several matches with short recovery periods between them (approximately 10 to 15 min), which may induce muscle fatigue and damage, reducing athlete performance over the competition. A previous study found decrements of up to ∼8–12% in isometric handgrip strength after the second match during an official judo competition (Kons et al., 2018c). In simulated judo matches, a 3.6% decrease was observed in countermovement jump (CMJ) performance after the second match and increased values of serum creatine kinase (CK) and lactate dehydrogenase (LDH) after the third match (Detanico et al., 2015), supporting that the physical effort expended during judo matches induced fatigue and muscle damage. Fatigue and muscle damage can also be caused by traditional training sessions. It is known that a 2-h judo-specific training session provokes increases in biochemical markers of muscle damage (CK and LDH) and in acute immune response (Umeda et al., 2008). In addition, Detanico et al. (2017a) reported increases in CK, LDH, and muscle soreness, as well as a decrease in lower limb performance (i.e., CMJ) 48 h after a traditional judo training session. However, upper limb performance recovered faster, returning to baseline values after 24 h, which indicates that lower limbs are more affected during judo practice.

High-intensity exercises, especially those involving short recovery time and high eccentric-concentric contractions (e.g., stretch-shortening cycle – SSC) induce immediate and prolonged reductions in muscle function due to fatigue and/or muscle damage (Nicol et al., 1996; Horita et al., 1999). The optimization of SSC seems to be a specific characteristic of judo training, since a previous study found better utilization of SSC in advanced judo athletes and higher CMJ height compared to untrained males (Zaggelidis et al., 2012). Moreover, CMJ performance is positively related to the number of throws in a judo-specific test (Detanico et al., 2012) and technical-tactical parameters in official judo competitions (Kons et al., 2018b). Since the efforts involving SSC generate high mechanical load, producing great stress in muscle structures (Nicol et al., 1996; Horita et al., 1999), strategies that attenuate exercise-induced fatigue would therefore maintain physical performance in combat and training sessions, allowing execution of repeated movement with greater intensities for longer periods. Moreover, reducing fatigue and muscle damage would allow faster recovery intra or between training sessions and between the combat sequences, thus acutely maintaining or chronically increasing physical performance and diminishing the risks of injury (Howatson and Van Someren, 2008).

Evidence has shown that photobiomodulation therapy (PBMT) prior to exercise can be used as a tool to attenuate fatigue in humans following strength and aerobic exercises (Borsa et al., 2013; Leal-Junior et al., 2013; Ferraresi et al., 2016; Rossato et al., 2017; Dellagrana et al., 2018a, b; Lanferdini et al., 2018a, b; Vanin et al., 2018). Moreover, it has recently been reported that PBMT reduced fatigue and facilitated faster recovery in another combat sport (i.e., Brazilian jiu-jitsu) (Araújo et al., 2017; Follmer et al., 2018). In summary, PBMT-related muscle fatigue attenuation mechanisms involve factors such as absorption of photons by chromophores and subsequent transduction of light energy into chemical energy within the cytoplasmic organelles (Reddy, 2004). In addition, increases in permeability and consequent transport by the cytoplasmic membrane have been observed (Klebanov et al., 2001), improving activity of oxidative enzymes associated with the IV complex (Oxidase C Cytochrome) (Silveira et al., 2009; Huang et al., 2011), and increased mitochondrial size and number (Manteǐfel and Karu, 2005). Considering muscle damage symptoms, several human trials have reported the benefits of applying PBMT prior to exercise to attenuate damage markers, such as delayed onset muscle soreness, strength impairments, and/or echo intensity increases (Baroni et al., 2010a; Antonialli et al., 2014; dos Reis et al., 2014; Fritsch et al., 2016). PBMT acts by reducing fatigue, as abovementioned, leading to improved ATP production and delaying cellular acidosis and its negative effect on cell metabolism (Hayworth et al., 2010; Karu, 2010). A reduction in reactive oxygen species and oxidative stress have also been observed, resulting in an anti-inflammatory action, which is related to muscle damage (Liu et al., 2009).

Athletes require attenuation of fatigue to maintain physical performance for longer periods, supporting high loads during training, high-intensity actions during competition, and reducing the risks of injuries (Abd-Elfattah et al., 2015; Takito et al., 2019). Moreover, they require attenuated muscle damage following training sessions and matches to accelerate recovery, which may result in maintenance of physical performance for the following sessions/matches (Nicol et al., 1996; Horita et al., 1999; Howatson and Van Someren, 2008). Therefore, PBMT may be an effective tool to attenuate judo athletes’ exercise-related fatigue and muscle damage in high-intensity efforts, as observed in judo matches and training sessions, maintaining performance and accelerating muscular recovery. Distinct sports can elicit different types of training-related physiological adaptations that are associated with their specificity, which could affect the athletes’ responses to PBMT. Although PBMT has been demonstrated to be effective for several sports and exercise modalities (Borsa et al., 2013; Leal-Junior et al., 2013; Ferraresi et al., 2016; Rossato et al., 2017; Dellagrana et al., 2018a, b; Lanferdini et al., 2018a, b; Vanin et al., 2018), no studies have investigated their effects in judo athletes. Thus, the purpose of this study was to investigate the effects of pre-exercise PBMT on fatigue and muscle damage markers (i.e., CMJ performance, muscle echo intensity, and soreness) up to 48 h after exercise in judo athletes. The initial hypothesis was that PBMT would attenuate fatigue-related reductions of CMJ performance, and diminish the increases in echo intensity and muscle soreness following exercise.

## Materials and Methods

### Participants

Athlete selection was performed in the east of Santa Catarina state, Brazil. The following inclusion criteria were adopted: (1) more than 6 years of judo practice; (2) graduation of purple, brown, or black belt athletes; (3) not be in a rapid weight loss or competition period; and (4) not presenting any musculoskeletal injury in the previous 2 years that could limit neuromuscular testing. Athletes were excluded according to the following criteria: (1) alcohol or medication intake during the data collection period; (2) not properly executing the neuromuscular assessments; (3) not reporting to the laboratory at the correct time of day; and (4) feeling any exacerbation (i.e., injury-related discomfort during the neuromuscular assessment).

Twenty-four judo athletes volunteered, of which sixteen completed all the study procedures. These comprised four purple belts, five brown belts, and seven black belts with the following characteristics: 23.1 ± 3.8 years, 77.9 ± 14.9 kg, 173.1 ± 8.9 cm, 17.5 ± 7.3% of body fat, and time of practice of 12.9 ± 5.0 years. All athletes had already participated in several national and state tournaments and were engaged in regular training (technical–tactical, aerobic, and resistance training) 3–4 times a week. The selected athletes were in the preparatory phase and therefore the athletes were not in a period of rapid weight loss. In addition, the participants were instructed not to intake alcohol or medication (e.g., anti-inflammatory or pain relievers) for at least 48 h before and during the evaluations, and to maintain their normal diet. Eight athletes were excluded due to previous lower limb injury (*n* = 3), participation in official competitions (*n* = 3), or a weight loss period (*n* = 2) during the data collection. Before the assessments, all subjects were informed about the procedures and signed an informed consent form. Ethical approval was obtained from the local Human Research Ethics Committee (30495314000005347), in accordance with the Declaration of Helsinki.

### Experimental Design

**FIGURE 1:**
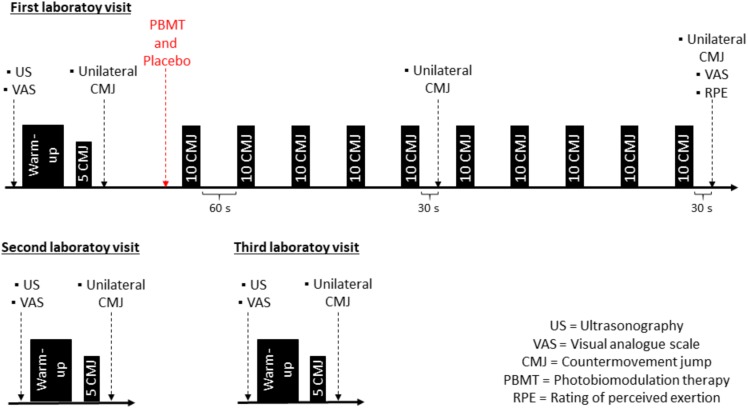
Experimental design of visits 1, 2, and 3.

This study was a randomized, triple-blinded, and placebo-controlled trial. After recruitment, 2 weeks before the experimental protocol, participants were familiarized with the CMJ (3 times/week) during their judo training sessions. The familiarization consisted of five bilateral and five unilateral (each leg) CMJs per day. Thereafter, participants were requested to avoid any lower limb exercises for 48 h before and during the 3 days of the experimental protocol (Figure 1).

In the first laboratory visit, body mass, height, and anthropometric measurements were recorded. Baseline measurements began with ultrasonography assessments. Thereafter, participants responded to a visual analog scale of muscle soreness and performed the warm-up and unilateral CMJ records. After baseline assessments, simple randomization was used to determine which lower limb would be treated with PBMT or placebo, to reduce any possible influence of limb preference on muscle fatigue and damage results. Eight participants received PBMT in the preferred limb (self-reported by each athlete) and eight in the non-preferred limb, whereas, the contralateral limb received placebo. In sequence, the plyometric protocol was performed to induce fatigue and muscle damage. After the fifth and after the tenth sets, one unilateral CMJ was performed for each leg. After each set, the rate of perceived exertion (RPE) was recorded, and after the complete protocol, the visual analog scale for fatigue and muscle soreness was applied.

The second and third laboratory visits took place 24 h (±2) and 48 h (±2) after the first visit, respectively. The visual analog scale of muscle soreness, ultrasonography, and CMJ assessments were performed exactly as described on the first visit, in the baseline assessments, before the PBMT application.

### Muscle Fatigue and Damage Protocol

To induce fatigue and muscle damage an SSC protocol was adopted, consisting of 10 sets of 10 repetitions of consecutive maximal bilateral CMJs (1-min rest between sets) on a piezoelectric force platform (model 9290AD; Kistler, Quattro Jump, Winterthur, Switzerland), which measures vertical ground reaction force at a sampling rate of 500 Hz. This protocol was recently adopted to investigate PBMT effects on muscle damage (Fritsch et al., 2016) and a similar protocol (30-s rest between sets) was used to induce fatigue. Participants received standardized instructions, verbal guidance, and encouragement during the protocol and the same researcher controlled all protocols. Participants were required to bend their knees and hips until approximately 90° of flexion in every CMJ. Fatigue was observed during the protocol due to a reduction of −9.5 ± 6.6% in mean power output and a rate of perceived exertion (0–10) of 6.4 ± 2.2.

### Countermovement Jump Assessment

Neuromuscular status, assessed through CMJ (usually performed bilaterally), has been used as a reliable non-invasive method to assess fatigue and muscle damage effects on performance (Wiewelhove et al., 2015; Claudino et al., 2017). In addition, the bilateral CMJ was correlated with technical-tactical variables (e.g., number of attacks, effectiveness, and effective combat time) obtained in official judo matches and judo-specific test performance (Kons et al., 2018b).

The CMJ was assessed using a strain gauge force platform (AMTI model OR6-6, Watertown, MA, United States), which measures vertical ground reaction force at a sampling rate of 1000 Hz. For the baseline, 24 h, and 48 h measurements participants performed a warm-up, consisting of 5 min on a cycle ergometer, 10 hops, five submaximal bilateral and three submaximal unilateral (each leg) CMJs (30-s rest between jumps). Two minutes later, five consecutive maximal CMJs were performed 30 s before the unilateral CMJs to ensure potentiated performance. To avoid exacerbated recovery, only one unilateral CMJ for each leg was recorded 30 s after the fifth and tenth sets of the plyometric protocol for fatigue monitoring. CMJs always began with the preferred limb, since PBMT was randomized. Half of the participants began with the PBMT treated limb and the other half started with the placebo treated limb.

For a proper technique, the participants started from a static standing on one foot position and were instructed to perform a countermovement (descent phase), followed by a rapid and vigorous extension of the lower limb joints (ascent phase) unilaterally. Their hands remained on their hips and they were instructed to jump as high as possible. Participants were allowed to land with both limbs to avoid exacerbated force on joints of a single limb landing. CMJ impulse (Kirby et al., 2011), peak power output, peak velocity, and peak force in the concentric phase of the jump were used for analysis (Cormie et al., 2008).

### Echo-Intensity

Muscular echo intensity, assessed through ultrasonography images, has been used as a reliable non-invasive method to assess the muscle damage level in humans (Chapman et al., 2011; Bottaro et al., 2012). Echo intensity of *rectus femoris* and *vastus lateralis* muscles was assessed using ultrasonography equipment (Logiq S7, GE Healthcare, Milwaukee, WI, United States) along with a linear array probe (50 mm, 5–15 MHz, ML6-15) from the same manufacturer, coated with a water-soluble transmission gel to provide acoustic contact without depressing the dermal surface.

Prior to each assessment, participants remained in the supine position with knees and hips in neutral position and rested for 10 min in order to allow fluid shifts to occur (Lopez et al., 2019). Three transversal images from the *rectus femoris* and *vastus lateralis* were obtained at 50% of the distance between the lateral condyle of the femur and the great trochanter (Korhonen et al., 2009). To ensure that all measurements were performed at the same site, on the first day a waterproof pen was used to mark the exact site where the subsequent assessments should be carried out. All measurements were performed by the same trained researcher.

Posteriorly, all images were analyzed with ImageJ software (National Institutes of Health, United States). A square with an area of 1 cm^2^ was positioned at the mid-point of the *rectus femoris* and *vastus lateralis* muscles for the echogenicity calculation (echo intensity). Echo intensity of each muscle was determined using the gray-scale analyses function and expressed in arbitrary units as a value between 0 (black) and 255 (white). The mean value between the three images from each day was used for analysis.

### Rate of Perceived Exertion, Rate of Perceived Fatigue, and Muscle Soreness Assessments

A visual analog scale was used to identify the occurrence of muscle soreness related to delayed onset muscle soreness at baseline, immediately after, and 24 and 48 h after the end of the protocol. The scale ranged from 0 to 10, in which 0 was considered the absence of soreness and 10 the presence of unbearable soreness. Participants reported muscle soreness in specific muscle groups of the lower body (knee extensors, knee flexors, and plantar flexors), by indicating the area on an anatomical diagram (Detanico et al., 2017b). The delayed onset muscle soreness after a high intensity effort is considered a marker of muscle damage (Howatson and Van Someren, 2008).

Rate of perceived exertion was recorded on a scale ranging from 0–10 (0 = no exertion at all and 10 = maximal exertion) after every set of the plyometric protocol. In addition, after the complete protocol participants indicated their perception of fatigue during the exercise for each leg using visual analog scales (Kuys et al., 2011), which consisted of a single 100 mm horizontal line with a headline statement at the top. The extreme left of the line indicated no fatigue, and to the extreme right, the statement indicated very fatiguing. Participants were asked to indicate their perception of fatigue with a single vertical line for each leg.

### Photobiomodulation/Placebo Treatment

Photobiomodulation therapy or placebo treatments were applied using a Chattanooga Intelect Mobile Laser 2779 system (Chattanooga Group, Guildford, Surrey, United Kingdom). PBMT or placebo were applied on 15 sites on each lower limb: eight sites on the quadriceps (three sites on *vastus lateralis*, three sites on *rectus femoris*, and two sites on *vastus medialis*), four sites on the hamstrings (two on *semitendinosus* and two on *semimembranosus*), two sites on the *gastrocnemius* (one on lateralis and one on mediallis areas), and one site on the *soleus*. Only the researcher responsible for the PBMT application was aware of the treatment. The other researchers that performed the assessments and participants were blinded to the respective treatments.

The PBMT/placebo treatment lasted about 8 min. While one lower limb received the PBMT, the other received the placebo simultaneously with two probes held stationary with skin contact at a 90° angle with light skin pressure. The placebo probe remained turned off throughout the treatment time. The PBMT general characteristics comprise cluster size = 30.2 cm^2^, number of sites = 15, treatment time per site = 32 s, dose per site = 30 J, and total dose = 450 J (Quadriceps = 240 J, Hamstrings = 120 J, and Gastrocnemius = 60 J). Recently a meta-analysis reported effective doses of 60–300 J for large and 20–60 J for small muscle groups (Vanin et al., 2018). Additional PBMT parameters are described in Table 1.

**TABLE 1:** Photobiomodulation therapy parameters.

**Parameters**	**LASERs (850 nm)**	**LEDs (670 nm)**	**LEDs (880 nm)**	**LEDs (950 nm)**
Number of diodes	5	12	8	8
Power output (mW)	100	10	25	15
Spot size (cm^2^)	0.06	1.92	1.28	1.28
Power density (mW/cm^2^)	1666.6	5.2	1.93	11.71
Frequency	Continuous	Continuous	Continuous	Continuous
Dose (J)	3.2	0.3	0.8	0.5

### Statistical Analysis

All statistical analyses were performed by a blinded researcher, who did not know which treatment groups “1” and “2” received. After analyses, and table and figure production, the groups were revealed and the respective labeling was included. The sample size was calculated (G^*^POWER software, version 3.1.9.2., Universität Kiel, Germany) for ANOVA repeated measures, within-between interaction (effect size = 0.25, α = 0.05, β = 0.80, number of groups = 2, number of measurements = 3, correlation among repeated measures = 0.5, non-sphericity correction = 1). A minimum of 14 participants per group was determined.

The intraclass correlation coefficient (ICC) and typical error as coefficient of variation (CV) were calculated for the three unilateral CMJ trials and for the echo intensity obtained from the three images for each muscle at baseline to verify their reliability (Hopkins, 2000). The normality of the distribution and homoscedasticity for outcome measures were tested using the Shapiro–Wilk, and Mauchly and Levene criteria, respectively. The Student *t*-test for independent samples was used to compare baseline of the preferred and non-preferred limbs for CMJ variables and echo intensity, as well as to compare the post-SSC protocol, VAS (fatigue), and the fatigue index between PBMT and placebo for CMJ variables. Two-way ANOVA repeated measures was used to analyze: (a) treatment (PBMT × placebo) × time (baseline, middle protocol, post-protocol, 24 h, and 48 h) interaction for unilateral CMJ variables; (b) treatment (PBMT × placebo) × time (baseline, post-protocol, 24 h, and 48 h) interaction for muscle soreness; and (c) treatment (PBMT × placebo) × time (baseline, 24 h, and 48 h) interaction for echo intensity. When Mauchly’s test of sphericity was significant and the Greenhouse-Geisser level of violation was >0.75, degrees of freedom were corrected using the Huynh-Feldt adjustment, and when violation was <0.75, the Greenhouse-Geisser correction was used. When a significant *F*-value was achieved, Bonferroni’s *post hoc* tests were used to determine the pair-wise differences between the different time points and between groups. An alpha level of 5% was used in all statistical analyses. Effect size was used to quantify the meaningfulness of any differences and was calculated using partial eta squared (η_p_^2^; trivial, <0.1; small, 0.1–0.29; moderate, 0.3–0.49; or large, ≥0.5) (Hopkins et al., 2009). ANOVA degrees of freedom are reported as df.

## Results

### Baseline Characteristics

**FIGURE 2:**
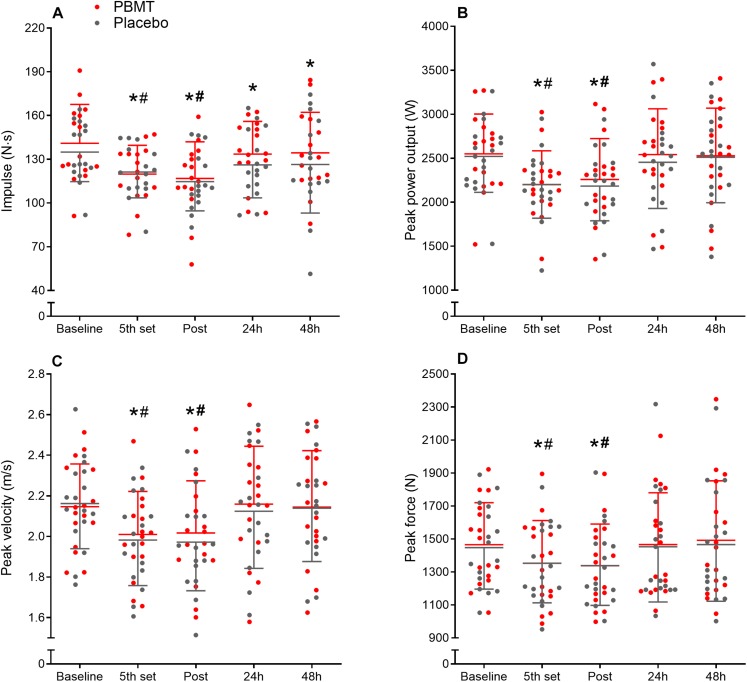
Countermovement jump impulse **(A)**, peak power output **(B)**, peak velocity **(C)**, and peak force **(D)** means and standard deviation at baseline, fifth set, immediately post, and after 24 and 48 h for PBMT (red lines) and placebo (gray lines). ^*^Different compared to baseline; #Different compared to 24 and 48 h.

Similar values were observed for preferred and non-preferred limb echo intensity (*rectus femoris* and *vastus lateralis*), and CMJ performance (peak force, peak velocity, peak power output, and impulse) (Table 2) at the baseline condition. In addition, the same was observed when comparing baseline values between PBMT or placebo treated limbs for all the abovementioned variables (Figures 1, 2), showing homogeneity of data for both legs and both conditions at baseline.

**TABLE 2:** Mean and standard deviation values (95% confidence interval upper and lower limits) for echo intensity and countermovement jump characteristics at the baseline.

**Variables**	**Preferred limb**	**Non-preferred limb**	***p-*value**
EI_RF_ (a.u.)	124.1±14.1⁢(116.6-131.6)	125.9±10.9⁢(120.1-131.7)	0.685
EI_VL_ (a.u.)	118.4±12.6⁢(111.7-125.2)	109.2±14.9⁢(101.3-117.2)	0.069
CMJ_PF_ (N)	1,438±239⁢(1,305-1,570)	1,415±222⁢(1,291-1,538)	0.793
CMJ_PV_ (m/s)	2.18±0.25⁢(2.05-2.32)	2.09±0.17⁢(2.00-2.19)	0.253
CMJ_PPO_ (W)	2,547±409⁢(2,320-2,773)	2,426±379⁢(2,216-2,636)	0.460
CMJ_IMP_ (N⋅s)	140±24⁢(126-153)	136±23⁢(123-149)	0.659

*EI, echo intensity; RF, *rectus femoris*; VL, *vastus lateralis*; CMJ, countermovement jump; PPO, peak power output; MPO, mean power output; PF, peak force; PV, peak velocity; IMP, impulse.*

### Countermovement Jump

There were no treatment-time interactions for CMJ impulse (df = 2.905; *F* = 0.690; *p* = 0.556; η_p_^2^ = 0.024), peak power output (df = 2.206; *F* = 0.279; *p* = 0.779; η_p_^2^ = 0.009), peak velocity (df = 2.108; *F* = 0.185; *p* = 0.842; η_p_^2^ = 0.006), and peak force (df = 1.631; *F* = 0.088; *p* = 0.880; η_p_^2^ = 0.003). While a moderate time effect was observed for impulse (df = 2.905; *F* = 15.009; *p* < 0.001; η_p_^2^ = 0.349) and peak power output (df = 2.206; *F* = 21.348; *p* < 0.001; η_p_^2^ = 0.416), and small time effect for peak velocity (df = 2.108; *F* = 8.456; *p* < 0.001; η_p_^2^ = 0.220) and peak force (df = 2.631; *F* = 11.497; *p* < 0.001; η_p_^2^ = 0.277).

Countermovement jump impulse reduced after the fifth set (*p* < 0.001) and at the end of the SSC protocol (immediately post, *p* < 0.001). Thereafter, it increased after 24 and 48 h compared to post (*p* < 0.014), but remained lower than baseline (*p* < 0.05) (Figure 2A). CMJ peak power output, peak velocity, and peak force reduced after the fifth set (*p* < 0.001) and at the end of the protocol (immediately post, *p* < 0.001), however, increased after 24 and 48 h (*p* < 0.006), returning to baseline values (*p* > 0.430) (Figures 2B–D, respectively). In addition, the fatigue index was similar between PBMT and placebo for CMJ impulse (*p* = 0.788), peak power output (*p* = 0.533), peak velocity (*p* = 0.318), and peak force (*p* = 0.560).

### Echo Intensity

**FIGURE 3:**
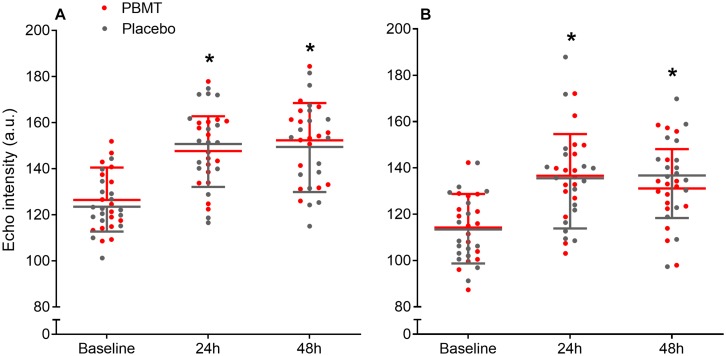
Mean and standard deviation for *Rectus femoris* **(A)** and *Vastus lateralis* **(B)** echo intensity at baseline, and after 24 and 48 h, for PBMT (red lines) and placebo (gray lines). ^*^Different compared to baseline.

There was no treatment-time interaction for echo intensity for either the *rectus femoris* or *vastus lateralis* (df = 2; *F* = 1.368; *p* = 0.262; η_p_^2^ = 0.044; and df = 1.947; *F* = 0.877; *p* = 0.419; η_p_^2^ = 0.028; respectively), while a large time effect was observed for both (df = 2; *F* = 96.911; *p* < 0.001; η_p_^2^ = 0.764; and df = 1.947; *F* = 49.155; *p* < 0.001; η_p_^2^ = 0.621; respectively). *Rectus femoris* and *vastus lateralis* echo intensity increased after 24 h (*p* < 0.001) and 48 h (*p* < 0.001), whereas 24 h was similar to 48 h (*p* > 0.327) (Figures 3A,B, respectively).

### Perception of Fatigue and Muscle Soreness

**FIGURE 4:**
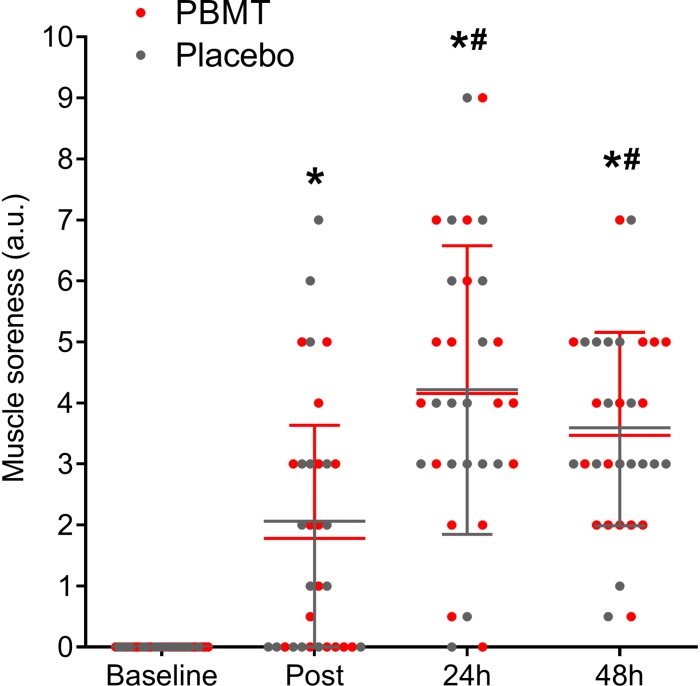
Quadriceps muscle soreness means and standard deviation at baseline, immediately post, and after 24 and 48 h for PBMT (red lines) and placebo (gray lines). ^*^Different compared to baseline; #Different compared to post.

There was no treatment-time interaction for quadriceps muscle soreness (df = 3; *F* = 0.046; *p* = 0.987; η_p_^2^ = 0.002) while a large time effect was detected (df = 3; *F* = 44.515; *p* < 0.001; η_p_^2^ = 0.597). Muscle soreness increased after the protocol (immediately post, *p* < 0.001) and increased again after 24 h (*p* < 0.001) and remained large after 48 h (*p* < 0.011) (Figure 4).

There was no difference (*p* = 0.532) in the visual analog scale for perception of fatigue between the placebo (62.0 ± 21.4, 95% CI: 50.7–73.4) and PBMT (57.4 ± 20.1, 95% CI: 46.7–68.1).

### Data Reliability

Reliability ranged from extremely high (>0.99) to high (>0.75 and <0.90) for the repeated measures of echo intensity and CMJ variables at baseline (Table 3).

**TABLE 3:** Baseline data reliability and typical error (95% confidence interval upper and lower limits).

	**Preferred limb**		**Non-preferred limb**	
	**ICC**	**CV %**	**ICC**	**CV %**
EI_RF_	0.993 (0.985–0.997)	1.04 (0.84–1.45)	0.986 (0.969–0.994)	1.10 (0.88–1.53)
EI_VL_	0.996 (0.990–0.998)	0.80 (0.64–1.10)	0.998 (0.996–0.999)	0.67 (0.54–0.93)
CMJ_PF_	0.949 (0.888–0.979)	4.83 (3.87–6.77)	0.978 (0.951–0.991)	2.91 (2.33–4.06)
CMJ_PVEL_	0.786 (0.581–0.906)	5.69 (4.55–7.98)	0.916 (0.820–0.965)	2.77 (2.22–3.86)
CMJ_PPO_	0.923 (0.834–0.968)	6.14 (4.90–8.61)	0.976 (0.947–0.990)	3.14 (2.51–4.38)
CMJ_IMP_	0.905 (0.790–0.962)	6.52 (5.22–9.51)	0.957 (0.900–0.983)	4.24 (3.40–6.15)

*EI, echo intensity; RF, *rectus femoris*; VL, *vastus lateralis*; CMJ, countermovement jump; PPO, peak power output; MPO, mean power output; PF, peak force; PV, peak velocity; IMP, impulse; ICC, intra-class correlation coefficient; CV, typical error as % of coefficient of variation.*

## Discussion

This was the first study to investigate the effects of PBMT on lower limb fatigue and muscle damage of judo athletes. Our initial hypotheses supposed that PBMT applied before exercise would be effective to attenuate fatigue-related reductions in CMJ performance, and thereafter reduce muscle damage symptoms after an SSC protocol. However, our main findings did not support any of the initial hypotheses, suggesting that PBMT was not effective to reduce fatigue and muscle damage in the lower limbs of judo athletes. These results counteract the findings observed in previous studies with non-athlete populations and different exercise modalities that will be described in the discussion.

The SSC protocol adopted in the present study successfully induced fatigue in lower limb muscles, as evidenced by decreased CMJ impulse, peak power, peak velocity, and peak force performance, and higher perception of fatigue. In summary, muscular fatigue can occur through ATP breakdown and accumulation of fatiguing substances (e.g., H^+^, Na^1+^, K^1+^, Ca^2+^ ions) without enough recovery time for their resynthesizes and removal (respectively) between exercise bouts, reducing contraction performance. It was expected that PBMT would attenuate fatigue through absorption of photons by chromophores and subsequent transduction of light energy into chemical energy within the cytoplasmic organelles (Reddy, 2004); increasing permeability and consequent transport by the cytoplasmic membrane (Klebanov et al., 2001), activity of oxidative enzymes associated with the IV complex (Oxidase C Cytochrome) (Silveira et al., 2009; Huang et al., 2011), and mitochondrial size and number (Manteǐfel and Karu, 2005). Moreover, the PBMT can increase microcirculation in a nitric oxide synthase–dependent mechanism, increasing the ATP/adenosine diphosphate (ADP) ratio and local arterial blood flow (Larkin et al., 2012). However, both PBMT and placebo treated limbs presented a similar decay through the SSC protocol, which indicates that PBMT had no positive effect to attenuate fatigue.

Our results are in disagreement with previous studies in which PBMT reduced fatigue following high intensity neuromuscular exercise. We could hypothesize that the observed differences may be due to the investigated population since some studies adopted non-athlete participants, which would explain the opposed results (de Souza et al., 2016; Rossato et al., 2016, 2017). However, other studies observed positive effects of PBMT in competitive athletes (e.g., Brazilian Jiu-Jitsu, volleyball, soccer, and cycling) (Ferraresi et al., 2015; Maldonado et al., 2015; Araújo et al., 2017; Follmer et al., 2018; Lanferdini et al., 2018b). Two studies that investigated PBMT effects in Brazilian Jiu-Jitsu athletes reported reduced upper limb fatigue. These positive effects were observed during an isometric elbow flexion (Follmer et al., 2018) or hand-grip strength (Araújo et al., 2017), which indicates that contraction mode, or the assessed limbs may be differently affected by PBMT and could potentially explain the disagreement with our study. For example, some studies adopted isokinetic dynamic contractions for knee extensors/flexors (Baroni et al., 2010b; de Brito Vieira et al., 2014; Rossato et al., 2017) or plantar flexors (de Souza et al., 2016), while others investigated isometric contractions (Rossato et al., 2016; Follmer et al., 2018). Isometric or isokinetic tests allow control of the angular velocity (e.g., 0–180°/s) and involve a single joint, differently from judo lower limb technique movement, which involves multi-joint explosive SSC contractions (Franchini, 2013). In addition, CMJ and maximum voluntary isometric contraction have demonstrated dissociated time courses of recovery and are possibly mediated by different mechanisms (Kennedy and Drake, 2018). The importance of SSC performance is evidenced by the relationship of CMJ performance with technical-tactical and specific test performance of judo athletes (Kons et al., 2018b). Thus, during competition combats or training sessions, muscle fatigue negatively affects judo athletic performance, and the adopted PBMT parameters seem to provide no benefit to attenuate these reductions.

Following a fatiguing high intensity exercise involving SSC, the presence of a great magnitude of muscle damage is expected, mainly due to the presence of eccentric actions (Nicol et al., 1996; Horita et al., 1999). The SSC protocol was effective in inducing muscle damage, as evidenced by decreased CMJ impulse, as well as increased *rectus femoris* and *vastus lateralis* echo intensity, and muscle soreness after 24 and 48 h. During exercises involving SSC, mechanical damage to muscular structures leads to disorganization of the Z-line, T-tubules, sarcoplasmic membrane and reticulum, myofibrils, and cytoskeletal. In the subsequent hours, alteration in Ca^2+^ homeostasis is observed, as well as induced inhibition of mitochondrial function, adenosine triphosphate depletion, inflammatory response, and proteolytic enzyme activation, increasing damage to muscle tissue (Cheung et al., 2003; Fredsted et al., 2007; Howatson and Van Someren, 2008). Attenuation in muscle damage was expected following the SSC protocol due to lower fatigue (Hayworth et al., 2010; Karu, 2010), which was not observed (as abovementioned). In addition, lower muscle damage was also expected due to a possible reduction in reactive oxygen species and oxidative stress, which result in an anti-inflammatory action, related to muscle damage (Liu et al., 2009).

The presence of muscle damage can decrease contraction performance and consequently reduce force and velocity of contraction (Howatson and Van Someren, 2008), as observed for CMJ impulse. This variable has a large correlation (*r*^2^ = 0.92) with jump height compared to peak power output (*r*^2^ = 0.45) and may better represent athletic performance (Kirby et al., 2011; Kons et al., 2018a). The similar decay in CMJ impulse, without differences between treatments 24 and 48 h after the SSC protocol, is a first indicative of the absence of a PBMT effect. Consequently, it is possible to suggest that PBMT cannot attenuate muscular function and, consequently, physical, technical, and tactical performance reductions following training sessions or combats in judo athletes. Our results are in agreement with a previous study that induced muscle damage with an equal SSC protocol, but assessed neuromuscular function with a knee extensor maximum voluntary isometric contraction in non-athletes (Fritsch et al., 2016). Conversely, other studies that induced muscle damage in knee extensors through isokinetic eccentric contraction and assessed muscular performance with maximum voluntary isometric contraction reported positive effects for muscle recovery when PBMT was applied (Baroni et al., 2010a; Antonialli et al., 2014). Our findings cannot provide information to state if the type of exercise used for inducing muscle damage (plyometric vs. isokinetic vs. constant load), the investigated population (athletes and non-athletes), the study design (crossover vs. paired groups), or the different PBMT parameters are responsible for these controversial results.

Echo intensity accessed by ultrasonography presented similar behavior regarding muscle damage. It is suggested that increases in ultrasound echogenicity (i.e., echo intensity) is due to increments in the interstitial space between fibers as a result of connective tissue damage and inflammation, as well as muscle swelling or increase in plasma enzyme levels (Chen et al., 2011, 2013). In our study, both the PBMT and placebo limbs presented similarly increased echo intensity for *rectus femoris* and *vastus lateralis* muscles at 24 and 48 h after the SSC protocol. This is an additional indicative of similar muscle damage between both limbs, showing no positive effect of PBMT. Our initial hypothesis was supported by a previous study (Fritsch et al., 2016) that reported maintenance of echo intensity for the PBMT treated limb while increases were observed only for the placebo limb after an identical study design and SSC protocol. However, our results are contrary to theirs, and although the mechanisms underlying this difference cannot be explained with our methods, this is an indicative that judo athletes and non-athlete physically active people differently respond to PBMT with the intention of muscle damage protection following an SSC protocol. The effectiveness of PBMT to prevent an inflammatory response in judo athletes seems to have no positive response or cannot be detected through echo intensity levels in ultrasonography images.

The muscle damage observed by lower CMJ impulse and higher echo intensity levels is accompanied by a similar increase in muscle soreness 24 and 48 h after the SSC protocol for both PBMT and placebo limbs. Thus, if the muscle damage was attenuated, it was expected that muscle soreness would also reduce. Despite the absence of damage reduction, PBMT can modulate pain through its direct effect on peripheral nerves (Douris et al., 2006), which was not observed in our study. Contrary to our findings, a previous study reported positive results for muscle soreness when PBMT was applied before a knee extensor eccentric protocol performed in isokinetic dynamometry (Fritsch et al., 2016). Fritsch et al. (2016) observed non-statistical differences between PBMT and placebo limbs after a plyometric protocol equal to ours. However, the authors reported that the PBMT limbs presented up to 30% less muscle soreness than placebo limbs, suggesting clinical relevance despite the absence of statistical significance. Conversely, our findings showed trivial percentage differences between PBMT and placebo treatments at 24 and 48 h after the SSC protocol (0 and 2.8%, respectively). For athletes, a minimal analgesic effect would positively affect performance, however, no effect was observed regarding muscle soreness.

Our findings are contradictory to the recent literature regarding PBMT effects on muscle fatigue (Vanin et al., 2018) and damage (Fritsch et al., 2016). The different markers of muscle fatigue (CMJ variables and visual analog scale for fatigue) and muscle damage (CMJ impulse, echo intensity, and muscle soreness) showed similar behavior following the SSC protocol, which differed from Fritsch et al. (2016) who showed dissociated results for echo intensity and strength production. The mechanisms underlying these differences cannot be explained by our methods, thus further studies should focus on understanding how and why different populations (e.g., according to training level, age, and illness), different muscle groups (e.g., lower or upper limbs), methods of fatigue and damage induction (e.g., jumping, running, and eccentric based protocols), assessment of fatigue and damage markers (e.g., CMJ, and dynamic or isometric peak torque or rate of force development), and different PBMT parameters are differently affected by PBMT.

Our study has both strengths and limitations. The use of a randomized, triple-blind, placebo-controlled trial design, where each evaluator performed the same tests throughout data collection, gave our study internal validity and reduced evaluator bias. The sample size allowed enough power (β > 0.8) to avoid any type II statistical error. In addition, the use of PBMT and placebo applied in the contralateral limb evaluated on the same day avoided within-subject between-day performance variability and a repeated bout effect that would hide possible positive effects. However, our design did not include biochemical muscle damage blood marker monitoring (e.g., CK). In addition, a wide variety of PBMT parameters have been used to reduce muscle fatigue and damage; therefore, it is unclear if this result would be the same using different parameters to those adopted in our study. Thus, future studies should focus on understanding the ideal PBMT parameters for athletes in order to attenuate fatigue and improve recovery, avoiding sports-related disorders (e.g., overtraining and muscle injuries).

## Conclusion

Our findings suggest no effects of PBMT applied before exercise to reduce lower limb muscle fatigue assessed by CMJ impulse, peak power output, peak velocity, peak force, and perception of fatigue during an SSC protocol (10 sets of 10 CMJ) for judo athletes. Additionally, no effects of PBMT to attenuate muscle damage markers (i.e., CMJ impulse, echo intensity, and muscle soreness) were observed following the damaging protocol adopted. Thus, judo athletes did not benefit from PBMT (with the respective parameters) applied before exercise to attenuate fatigue and muscle damage symptoms during and following our SSC protocol.

## Ethics Statement

Ethical approval was obtained from the local Human Research Ethics Committee of the Federal University of Santa Catarina.

## Author Contributions

All authors conceived the study design, participated in the interpretation of data, drafted the manuscript, and read and approved the final version of the manuscript. LO, RK, RS, and JS carried out the data collection. LO carried out all the statistical analyses.

## Conflict of Interest Statement

The authors declare that the research was conducted in the absence of any commercial or financial relationships that could be construed as a potential conflict of interest.
